# Diversity of lncRNAs in the pan-transcriptome of maize inbred lines

**DOI:** 10.1186/s12864-025-12242-0

**Published:** 2026-01-03

**Authors:** Artem Yu. Pronozin, Nikolai A. Shmakov, Dmitry A. Afonnikov

**Affiliations:** 1 Institute of Cytology and Genetics, Siberian Branch of Russian Academy of Sciences, 630090 Novosibirsk, Russia; 2 Kurchatov Center for Genome Research, Institute of Cytology and Genetics, Siberian Branch of Russian Academy of Sciences, 630090 Novosibirsk, Russia; 3 Faculty of Natural Sciences, Novosibirsk State University, 630090 Novosibirsk, Russia

**Keywords:** Long noncoding RNA, Maize inbred lines, Pan-transcriptome, Evolution, Conservation, MRNA, SNP

## Abstract

**Background:**

Long non-coding RNAs (lncRNAs) constitute a substantial portion of the plant transcriptomes and performs important functions in numerous molecular, regulatory, growth and developmental processes and stress responses. However, functional characteristics supported by experimental evaluation are known only for a small part of lncRNAs. In this regard, evolutionary and comparative analysis of lncRNA sequences can provide additional information about the functional role of these molecules.

**Results:**

Analysis of RNA-seq libraries from 503 maize inbred lines obtained by Hirsch et al. (2014) enabled the assessment of sequence diversity and evolutionary patterns of maize lncRNAs within the pan-transcriptome framework and their comparison with analogous mRNA characteristics. The lncRNA pan-transcriptome comprises a greater number of representative sequences (595,198), compared to mRNA pan-transcriptome (245,436), smaller fraction of core and shell parts and larger cloud component (52.5% vs 11%). However, both pan-transcriptomes are closed according to estimates of the power-law parameters. Nucleotide diversity of the lncRNAs significantly higher compared to mRNAs. Moreover, nucleotide substitution rates estimates for coding and non-coding sequences demonstrated systematic increase of the gamma distribution shape parameter α in the order α(*K*_a_) < α(*K*_s_) < α(*K*_n_) across all pan-transcriptome components (core, shell, cloud). Comparison of evolutionary characteristics also demonstrated that antisense lncRNAs are the most conserved in terms of both nucleotide substitution rates and their representation in the core; intronic lncRNAs display the highest mutation rate, while intergenic lncRNAs exhibit the greatest sequence repertoire diversity and highest line specificity.

**Conclusions:**

These results allowed to evaluate the diversity of lncRNAS from the pan-transcriptomic point of view and supported their high lineage-specificity and sequence variation across maize inbred lines in comparison to mRNA sequences.

**Supplementary Information:**

The online version contains supplementary material available at 10.1186/s12864-025-12242-0.

## Introduction

Long noncoding RNAs (lncRNAs) represent a class of linear or circular RNA molecules exceeding 200 nucleotides in length that do not code for proteins. Similar to mRNAs, lncRNAs contain splicing sites, exon sequences, and a poly(A) tail [1]. Regulation of their expression occurs analogously to mRNA. However, it has been shown that their expression is highly tissue-specific and its average level is lower than that of mRNA [2, 3]. lncRNAs can be classified into three groups based on their genomic location relative to protein-coding genes [4, 5]: antisense (transcribed from the complementary DNA strand within coding gene regions), intronic (located within gene introns), and intergenic (located between protein-coding genes).

lncRNAs constitute a substantial portion of the transcriptome [6]; however, their functions were initially poorly understood, and these molecules were considered “transcriptional noise” [7]. Subsequent analysis revealed that lncRNAs participate in a wide range of critical processes in animals and plants: regulation of gene expression [8], formation of macromolecular complexes [9], interaction with proteins [10], and underlying the development of pathogenesis in animals and humans [11]. Compared to animals, the functions of lncRNAs in plants have been studied in less detail. Nevertheless, experimental evidences demonstrate their involvement in regulating resistance to cold, salt, and heat stress [12–15], influence on hypoxia tolerance [16], and participation in the development of fruits, roots, and leaves [17, 18], as well as numerous other plant growth and developmental processes and stress responses [19, 20]. Plant lncRNAs function as both genetic and epigenetic regulators of gene expression. They can act as cis-regulatory elements by interacting with neighboring genes or as trans-regulatory elements, influencing genes located distantly from their site of synthesis [21]. This functional diversity makes lncRNAs an important subject for understanding the molecular mechanisms of plant growth and development, and their response to biotic and abiotic stresses. However, to date, the functional role has only been experimentally validated for a small fraction of lncRNAs. For the majority of lncRNAs, their functions remain unknown.

Comparative analysis of sequences from genomes of diverse organisms can provide substantial insights into the functional roles of lncRNAs. According to current knowledge, functionally important sequences exhibit higher conservation. Studies of lncRNA evolution in animals [22, 23] and plants [24–26] have revealed several key features of these molecules. The number of identified plant lncRNAs per species correlates with genome size, with maize exhibiting the highest count among 26 analyzed species [24]. While the structural properties of lncRNAs (length distribution, exon number, and expression level relative to mRNAs) are remarkably similar across different plant species [24, 27], the number of shared lncRNAs is higher among closely related species (within the same genus). The proportion of shared lncRNAs decreases sharply with increasing evolutionary distance [24, 25]. Thus, the majority of lncRNAs are species-specific. Numerous studies have demonstrated high sequence variability in lncRNA primary structure compared to mRNA [7, 23, 28–30]. lncRNAs exhibit a higher proportion of single-nucleotide substitutions relative to mRNA [24]. In cross-species comparisons, antisense lncRNA sequences show the highest conservation, intergenic lncRNAs are more variable and intronic lncRNA sequences display the greatest variability [25, 26, 31].

Recent research on plant genomes has increasingly focused on pan-genome analysis, which refers to a non-redundant set of genes present across genomes within a taxonomic unit (typically a species) [32–36]. The pan-genome concept encompasses protein-coding gene sequences subject to presence-absence variations (PAVs) among multiple accessions of a species, some of which may be absent in the reference sequence. Pan-genome structure is generally divided into three main components: the core (conserved genes present in all accessions), the shell (genes present in a significant proportion of accessions), and the cloud (genes present in only 1–3 accessions) [32]. Analysis of pan-genome structure reveals functional differences among gene sequences belonging to its distinct compartments. The pan-genome core contains conserved genes performing fundamental biological functions. Genes associated with plant adaptation to environmental conditions (immune response and abiotic stress response) are predominantly classified within the shell and cloud parts [32, 33]. In some cases, the plant pan-transcriptome may serve as an approximation of the pan-genome. It is defined as genes expressed across a set of accessions of a species. This approach considers expression-based presence-absence variations (ePAVs). Nevertheless, structural features of pan-genomes and pan-transcriptomes show close correspondence [36–39].

Pan-genomes and pan-transcriptomes are divided into two categories, open and closed [32]. In open pan-genomes, size increases continuously with the addition of each new genome. In closed pan-genomes, beyond a certain number of accessions, adding new genomes will not result in the discovery of novel genes. The nature of a pan-genome can be determined using a power law relationship describing the proportion of novel genes added relative to the number of genomes analyzed [40].

Here, we use a pan-transcriptome approach to assess lncRNA diversity within maize. Five hundred three transcriptome libraries from maize inbred lines previously generated by [37] were used as the input data. Pan-transcriptomes encompassing both protein-coding genes and lncRNAs, were analyzed; their structural composition allowed identification of conserved and variable genes within both pan-transcriptomes. Identification and analysis of single-nucleotide polymorphisms (SNPs) in mRNAs and lncRNAs was performed to compare the distribution patterns of nucleotide substitutions. Additionally, synonymous and non-synonymous substitutions for protein-coding genes and lncRNAs were evaluated. As result, differences in the structure of mRNA and lncRNA pan-transcriptomes were identified, as well as distinctive patterns of nucleotide substitution accumulation in these two molecular classes.

## Materials and methods

### Transcriptomic datasets

Five hundred three transcriptome libraries of maize inbred lines from the study by [38] were used for analysis. Libraries were obtained from whole-seedling tissue including roots at the V1 stage (first leaf collar) [41]. RNA from three plants per inbred line was pooled and sequenced using 100-bp paired-end reads on the Illumina HiSeq platform [38]. The list of maize lines used and their corresponding libraries is provided in Supplementary Table S1. The maize genome sequence v.40 (AGPv4) [42] was used as reference. The annotation was retrieved from the Ensembl Plants database v.50 [43].

### Assembly of individual maize line transcriptomes

Quality control of the investigated libraries was performed using FastQC v0.11.8 [44]. Reads were filtered with FastP v0.22.0 [44] using the following parameters: average sequencing quality (Phred score) ≥ 20, minimum length = 50 bp, overlap length = 50 bp.

Transcriptome assembly was performed independently for each library using the Trinity, Trinity Genome-Guided (TrinityGG), and SPAdes tools. These tools were selected due to their high quality and accuracy in de novo transcriptome assembly [45–47]. For de novo assembly Trinity v2.9.1 [48] and SPAdes v3.13.0 [49] in the rnaSPAdes mode were used with default parameters. For reference genome-based transcriptome assembly, short reads were aligned to the maize reference genome using HISAT2 v2.2.1 [50]. The resulting mapping files were processed and sorted using SAMtools v1.6 [51]. The processed alignment files were then used as input for Trinity in Genome-Guided mode [52], with the maximum intron length constrained to 500,000 nucleotides.

### Pan-transcriptome assembly for coding sequences

For each of the three obtained assemblies per individual line, transcripts were partitioned into coding and non-coding components using ICAnnolncRNA [31]. This pipeline enables high-accuracy classification of transcripts into protein-coding and lncRNAs via the LncFinder method [53]. The pipeline's output categorizes transcripts from independent assemblies of individual maize lines into coding and non-coding sequences, which undergo subsequent analysis. Coding sequences from the three independent assemblies per library were processed using tr2aacds.pl from the EvidentialGene package [54] to remove redundancy and contamination. Expression levels of filtered transcripts were quantified with Kallisto v0.46.2 [55]; transcripts with low expression (TPM < 1) were excluded from further analysis. Amino acid sequences corresponding to the retained transcripts were generated using EvidentialGene. To eliminate contamination, amino acid sequences were compared with the NCBI UniVec database [56] using BLASTx v2.5.0 [57]. Significant hits were excluded from further analysis. Next, amino acid sequences were compared with those from the monocot species in the PLAZA database [58]: *Zea mays B73*, *Saccharum spontaneum*, *Sorghum bicolor*, *Oryza sativa ssp. indica*, *Oryza sativa Kitaake*, *Oryza sativa ssp. japonica*, *Brachypodium distachyon*, *Hordeum vulgare*, *Triticum dicoccoides*, *Triticum turgidum*, and *Triticum aestivum*. Sequences exhibiting < 90% similarity to PLAZA were discarded.

A meta-assembly was generated from remained mRNA sequences of the three assemblies per individual library. This approach compensates for individual assembler limitations while enhancing overall assembly quality [46, 59–62]. Library meta-assemblies were constructed using trformat.pl from the EvidentialGene package [54]. Meta-assembly sequences from individual libraries were consolidated into a pan-transcriptome representing the coding fraction of maize inbred lines. To establish gene composition, amino acid sequences were clustered using USEARCH v11 [63] at 80% identity threshold. Expression levels for resulting centroids were quantified with Kallisto v0.46.2 [55] across all maize libraries. Sequences in the clusters with TPM < 1 for centroids were removed from analysis.

### Pan-transcriptome assembly for noncoding sequences

LncRNA redundancy and contamination removal was performed in several steps. First, low-expression transcripts (TPM < 1) were removed using the ICAnnolncRNA pipeline. Second, for transcripts mapped to the reference genome, redundancy due to transcripts aligned to the same loci was eliminated and transcripts overlapping mobile elements were removed by ICAnnolncRNA [31]. Finally, lncRNA transcripts that aligned to exons or their parts in the same strand were removed from analysis because they may represent mRNA fragments occurring due to errors in transcriptome assembly. Only antisense, intronic or intergenic lncRNAs were used in subsequent analysis.

A substantial proportion of transcripts remained unmapped to the reference genome after alignment procedure. They were compared using BLASTn v2.5.0 [57] with known protein-coding genes, non-coding RNAs (mRNAs, microRNAs, tRNAs) from the Ensembl Plants database [64] and library of transposable elements of maize obtained in previous work [31]. Transcripts were removed if (1) comparison with protein-coding sequence yielded qcovs = 100% (percentage of query sequence covered relative to target) and sstrand = plus (the same strand orientation); (2) comparison with non-coding RNAs yielded qcovs = 100%; (3) identity with mobile element sequence was above 80%.

After filtration, non-coding transcripts from individual libraries were consolidated into a lncRNA pan-transcriptome. These sequences were clustered using VSEARCH v2.15.2 [65] to identify representative transcripts (centroids). When clustering lncRNAs, we took into account that even for the same pair of mRNAs the identities in nucleotide and amino acid sequences differ [66]. Therefore, applying the same similarity threshold (80%, see Sect. "Pan-transcriptome assembly for coding sequences"* Pan-transcriptome assembly for coding sequences*) both for lncRNA and amino acid sequence clustering would lead to differences in two pan-transcriptome structures due to inequality of divergence rates. Therefore, empirical procedure was performed to select clustering threshold for lncRNAs that roughly correspond to 80% similarity of amino acid sequences. 100 mRNA sequences from different clusters were chosen randomly. Those of them from the same clusters were aligned by MACSE v2 [67] to keep codon correspondence. Nucleotide dissimilarity was estimated for sequence pairs with amino acid dissimilarity close to 0, 10, 20, 30, and 40%. The resulted distribution are shown as bar plot in Supplementary Figure S1. The figure demonstrates that for 20% dissimilarity of protein sequences the third quartile of nucleotide dissimilarity distribution is close to 30% (median is 24.5%). Therefore, 70% identity threshold was used for lncRNA sequence clustering. As for protein-coding genes, clusters of lncRNA transcripts were eliminated from analysis if expression level of their centroids were below 1 TPM.

Thus, our pipelines represent similar methods of data processing and pan-transcriptome reconstruction both for mRNA and lncRNA transcripts for 503 maize inbred lines.

### Pan-transcriptome analysis

The pan-transcriptome analysis pipeline is shown in Supplementary Figure S2. Based on centroid expression, expression presence-absence variations (ePAVs) were identified in each maize inbred line. Plots depicting the size of the pan-transcriptome and core transcriptome in relation to the number of lines included were constructed using PanGP algorithm [68].

To separate sequences in the pan-transcriptome into core, shell and cloud we used following thresholds: core transcripts expressed in 100% of inbred lines; shell transcripts expressed in 10 to 90% inbred lines; cloud transcripts expressed in less than 10% inbred lines.

To determine the type of the pan-transcriptome we estimated power law parameters *α* and tg(*θ*) for the relationship between the number of novel transcripts added to pan-transcriptome and the number of libraries [40]. The *α* exponent determines the pan-genome/pan-transcriptome type: *α* ≤ 1 for open pan-transcriptome; *α* > 1 for closed pan-transcriptome. The second parameter, tg(*θ*), is the asymptotic increase in the number of novel genes in the pan-genome/pan-transcriptome upon adding a new representative.

### Pan-transcriptome SNP analysis

Estimates of the nucleotide diversity (*π*) and Tajima’s *D* parameters were used to evaluate the nucleotide diversity of mRNA and lncRNA sequences in inbred maize lines [69–71]. *π* is a measure of genetic diversity within a population. It indicates the level of nucleotide variation among individuals in the population. Higher *π* values correspond to greater nucleotide diversity and, hence, greater SNP variation. *D* statistics determines the extent to which the allele frequency distribution deviates from that expected under neutral evolution. When *D* ≈ 0, substitutions conform to the neutral evolution model. *D* > 0 indicates an excess of rare alleles compared to neutral evolution expectations, which may suggest balancing selection or the effect of a recent population expansion. *D* < 0 indicates a deficit of rare alleles relative to neutral evolution expectations, which may point to purifying (negative) selection.

*π* and *D* parameters were estimated using the vcftools v.0.1.16 [72], which utilizes genomic variation files as input. To generate these files, we performed reads mapping from individual libraries onto centroid sequences using bwa mem v.0.7.17 [73]. Polymorphism detection (SNPs, insertions, and deletions) was performed using a combination of samtools mpileup and bcftools [51]. The resulting polymorphism dataset underwent filtering based on MAPQ > 30, missing value ≤ 0.25, MAF ≥ 0.05, HWE ≤ 0.01, and LD ≤ 0.2. Transcript positions with substitutions passing these filters were used for *π* and *D* calculations. The Mann–Whitney test was performed to assess the statistical significance of differences between protein-coding genes and lncRNA groups.

### Analysis of synonymous and non-synonymous substitutions

To assess the evolutionary mode of substitutions in mRNA and lncRNA sequences in the population of inbred maize lines, we estimated the rates of non-synonymous (*K*_*a*_) and synonymous (*K*_*s*_) substitutions for mRNA and nucleotide substitutions (*K*_*n*_) for lncRNA. These parameters were evaluated using KaKs_calculator v3 [74], which requires pairwise nucleotide sequence alignments against reference sequences as input. Therefore, for analyzing substitutions in coding sequences, we used CDS sequences from the maize reference genome in the Ensembl Plants v.50 database [64].

For lncRNA analysis, sequences from PLncDB v.2.0 [75] were utilized. This database combines sequences from several lncRNA databases, including EVlncRNAs [76] (a database of experimentally validated lncRNAs), and assigns confidence levels to its entries. LncRNAs are thus classified by confidence level, ranging from low to high. Consequently, only lncRNAs from the EVlncRNAs database and those with high confidence levels were selected. Subsequently, transcript alignment for each of the 503 assemblies to reference CDS and lncRNA sequences was performed using GMAP [77].

The analysis was performed for individual library sequences. For mRNA, we evaluated the distribution of *K*_*a*_ and *K*_*s*_ rates and their ratio (*K*_*a*_/*K*_*s*_) for each transcript. For lncRNA, we assessed the distribution of the *K*_*n*_ parameter and its ratio to the mean *K*_*s*_ value across all mRNA transcripts in the transcriptome.

To evaluate the substitution mode in mRNA and lncRNA sequences, we fitted gamma distributions to the *K*_*a*_, *K*_*s*_, and *K*_*n*_ histograms, parameterized by shape (*α*) and rate (*β*) [78]. The first parameter (*α*) characterizes the substitution pattern: if *α* < 1, sequences are conserved and contain only a small fraction of sites with high substitution rates. If *α* > 1, the sequences exhibit sites with broad variation in substitution rates [78]. Comparisons of *K*_*a*_, *K*_*s*_, and *K*_*n*_ distributions were conducted between mRNA and lncRNA, as well as across sequences stratified by pan-transcriptome groups (core, shell, cloud) and types (antisense, intergenic and intronic).

## Results

### Pan-transcriptome assembly

**Fig. 1:**
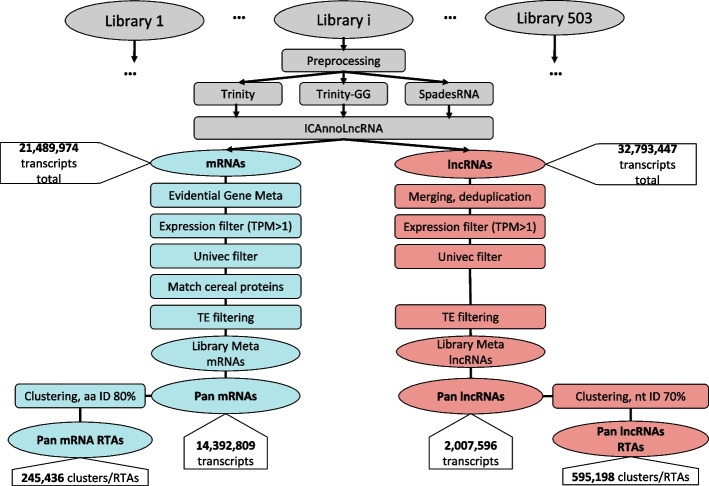
Pan-transcriptome analysis pipeline. Gray color denotes analysis stages common to both mRNA and lncRNA. Blue color designates mRNA-specific steps; red color indicates processing steps specific to lncRNA. This workflow identified 245,436 protein-coding gene centroids and 595,198 lncRNA centroids

The pan-transcriptome assembly pipeline and its partitioning into coding and non-coding components are illustrated in Fig. 1. Quality metrics for de novo transcriptome assemblies generated by three methods are provided in Supplementary Table S1. The highest number of transcripts (44,149,357) was obtained with the rnaSPAdes assembly, while the lowest number resulted from the reference-guided Trinity assembly. In general, de novo assemblers yielded much more transcripts in comparison with genome guided assembly (dozens of millions vs hundreds of thousands). Nevertheless, all three assemblers demonstrate high-quality assembly metrics that show strong concordance across methods (average values for N50 parameter are above 1,400).

Supplementary Table S2 shows results of transcript filtering by length (> 200 nt) and their classification into coding/non-coding types for assemblies obtained by three different methods. It demonstrates that all three assemblers generate sufficiently long sequences: more than 70% pass the length filter. However, the rnaSPAdes assembly generates more short transcripts compared to Trinity. The table further reveals that non-coding RNAs predominate in transcriptomes. These proportions vary slightly among assemblers: de novo algorithms produce a lower fraction of non-coding sequences than the reference-based approach.

Supplementary Table S3 shows results of filtering steps for mRNA transcripts. The number of protein sequences increases slightly after Evidential Gene processing by accounting for different mRNA isoforms. As recommended by the authors [54], we retained all variants produced by the software. Fraction of sequences remained after filtering by expression and similarity to Univec/viral sequences is similar for all methods, however fraction of sequences removed due to similarity to non-cereal proteins is larger for genome guided assembly (39% versus 17%).

Non-coding sequence processing statistics described in Supplementary Tables S4, S5 and S6. The largest decrease in the number of transcripts aligned to the reference genome occurred after redundancy removing for sequences aligned to the same genomic loci (see Supplementary Table S4 for details). Their number drops by more than 90%. Large decrease in the transcripts number (by ~ 70–50%) observed after removing transcripts overlapped with TEs. Application of different filters to lncRNA transcripts unmapped to reference genome (similarity to TEs, protein-coding and known ncRNAs) demonstrated gradual decrease of the remaining sequence number (by ~ 1–10%). Sharp decrease in sequence number (by 30 to 85%) observed after redundancy removal (see Supplementary Table S5 for details). Merging both mapped and unmapped filtered lncRNA yielded about 2 million transcripts. Contamination filter and deduplication result in only slight (by ~ 1%) decrease in sequence number for all types of assembly datasets (see Supplementary Table S6 for details).

The centroids resulting from clustering are designated as pan-transcriptomes for the coding and non-coding components. Their numbers provided in Supplementary Table S7 and Fig. 1.

### Pan-transcriptome structure analysis

**Fig. 2:**
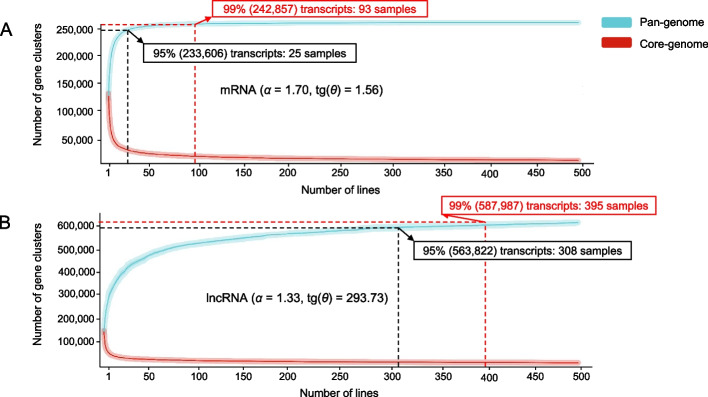
Pan-transcriptome structures of maize inbred lines for coding **A** and non-coding **B** sequences. The X-axis represents the number of maize inbred lines. The Y-axis displays the count of sequence clusters constituting the pan-transcriptome (blue line) and its core (red line). Graph insets provide power-law distribution parameters for each pan-transcriptome

Results of the pan-transcriptome structure analysis for coding and non-coding sequences are presented in Fig. 2. The figure demonstrates that gene counts in both pan- transcriptomes reach a plateau as the number of analyzed inbred lines increases. Thus, both exhibit a closed configuration, as evidenced by alpha (*α*) values > 1 for the power-law distribution. Nevertheless, significant differences between the two pan-transcriptomes are observed.

The analysis demonstrates that 25 inbred lines represented an average 95% the mRNA pan-transcriptome, while 93 lines covered 99% of this pan-transcriptome (Fig. 2A). The pan-transcriptome alpha parameter for lncRNA (1.33) is smaller than that for mRNA indicating its higher ability to acquire novel sequences. The lncRNA pan-transcriptome requires 308 inbred lines to represent an average 95% of its sequences, while 395 lines represented 99% of pan-transcriptome (Fig. 2A).

Notably, while the lncRNA pan-transcriptome expands more slowly, its core component size decreases faster than that of mRNA. After the 30th line addition, the lncRNA core stabilizes near 27,582 clusters (Fig. 2B), whereas the mRNA core demonstrates significant change approaching 19,798 clusters after ~ 60 lines (Fig. 2A). These results indicate greater diversity in the lncRNA pan-transcriptome compared to mRNA.

**Fig. 3:**
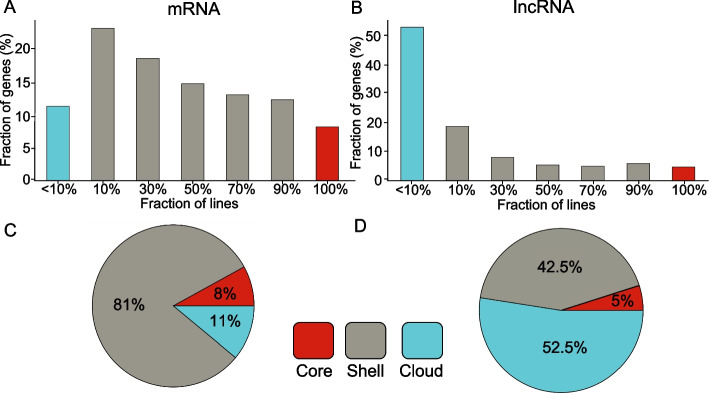
Distribution of genes across transcriptomes of coding **A** and non-coding **B** RNAs by the proportion of maize inbred lines sharing them. The X-axis indicates the fraction of maize lines (relative to total), while the Y-axis shows the percentage of RNAs present in those lines. Distribution of genes within core, shell, and cloud components of the pan-transcriptome for coding **C** and non-coding **D** RNAs

The distributions of mRNA and lncRNA transcripts from core, shell and cloud parts of the pan-transcriptome are shown in Fig. 3. For the mRNA pan-transcriptome, the core comprises 8% of genes, with most genes classified as shell. As the number of shared lineages decreases, the mRNA proportion increases (Fig. 3A), peaking at ~ 25% for genes present in 10% of lines. However, below 10% prevalence (cloud component), the mRNA proportion drops nearly twofold to ~ 12%. The fraction of common lncRNAs also increases steadily with decreasing the number of shared lines. However, its largest value, 52%, corresponds to genes present in less than 10% of lines (Fig. 3B).

Two pan-transcriptomes differ by fractions of genes belonging to core, shell and cloud parts (Fig. 3C,D). The proportions of lncRNA transcripts in the shell and core categories are nearly twofold lower than those of mRNA (42% vs. 81% for shell; 5% vs. 8% for core; Fig. 3C,D). On the contrary, lncRNA pan-transcriptome have large fraction of cloud transcripts, almost five times greater than for mRNA (52% vs 11%). These results confirm greater sequence diversity in the lncRNA pan-transcriptome compared to mRNA one.

Notably, the fractions of core, shell and cloud transcripts are very similar in individual transcriptomes with rare exceptions (see Supplementary Fig. S3). This holds true for both mRNAs and lncRNAs. However, average fraction of core, shell and cloud transcripts for single line differ markedly between mRNAs and lncRNAs. Individual protein coding transcriptome is characterized by 16% core, 83% shell and 1% cloud transcripts. Individual lncRNA transcriptome is characterized by 18% core, 75% shell and 7% cloud transcripts. Again, this pattern suggests lncRNA transcriptome diversity and volatility.

### Analysis of nucleotide diversity in pan-transcriptome

Variant calling and analysis within the maize pan-transcriptome for mRNA and lncRNA components initially identified 3,299,149 and 7,996,789 SNPs, respectively. Following quality filtering (see Materials and Methods, Sect. "Pan-transcriptome analysis"), 3,116 high-confidence SNPs remained for lncRNA and 2,743 for mRNA.

**Fig. 4:**
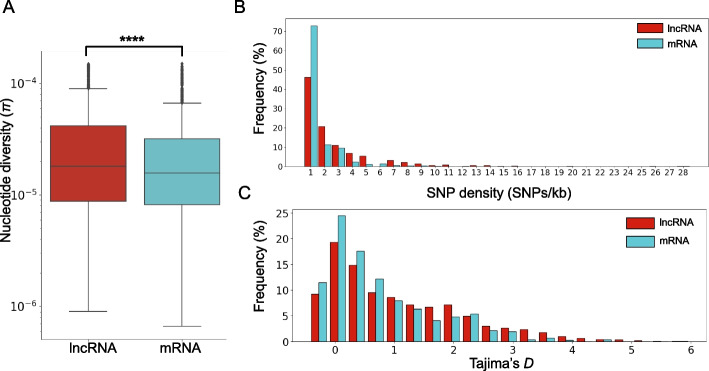
Nucleotide diversity analysis of coding and non-coding RNAs in the maize pan-transcriptome. **A** Boxplots of nucleotide diversity (*π*) per 10 kb. Y-axis corresponded to log₁₀(*π*). **B** SNP density distribution histograms. X-axis corresponded to SNPs/kb and Y-axis to proportion of RNAs. **C** Tajima’s *D* distribution histogram per 10 kb. X-axis corresponded Tajima’s *D*, Y-axis to proportion of RNAs. LncRNA distributions are shown in red; mRNA in blue

Results of nucleotide diversity (*π*), SNP density, and Tajima's *D* distributions are presented in Fig. 4. Mean *π* values were 3.677 × 10⁻^5^ for lncRNA and 2.899 × 10⁻^5^ for mRNA (Fig. 4A), rejecting the null hypothesis of equality (*p* < 0.0001, Wilcoxon Signed Rank Test). Additional statistics of *π* distributions for mRNAs and lncRNAs are shown in Supplementary Table S8. Table demonstrates that median *π* is larger for lncRNAs (1.94 × 10⁻^5^) than for mRNAs (1.58 × 10⁻^5^). *π* distribution for lncRNAs has larger range than for mRNAs (interquartile range 3.77 × 10⁻^5^ vs 2.42 × 10⁻^5^). Thus, lncRNA sequences exhibit greater nucleotide variation than mRNA.

Comparison of SNP density distributions (Fig. 4B) reveals a shift toward lower values in mRNA: 60% of mRNA sequences show ≤ 1 SNP/kb, whereas this fraction drops to 40% for lncRNA.

Tajima’s *D* calculation for both sample types revealed a distribution skewed toward positive values (Fig. 4C). Negative values comprised 19% for mRNA and 17% for lncRNA, with no observations below −0.44. Sequences with *D* > 2 constituted 13% (mRNA) and 20% (lncRNA). These results suggest non-neutral evolution for a substantial fraction of genes (both coding and non-coding) characterized by an excess of rare alleles.

### Analysis of synonymous and non-synonymous substitutions

A sample of 72,539 reference CDS sequences of maize was used to estimate nucleotide substitution rates *K*_*a*_ and *K*_*s*_ in mRNA. A total of 8,163,024 mRNA sequences from the pan-transcriptome were aligned to CDS, representing 34% of all identified protein-coding genes (23,975,474; see Supplementary Table S3). Among these, 4,222 were classified as core, 16,664 as shell, and 1,174 as cloud. This yielded 6,201,766 nucleotide substitutions in protein-coding genes.

The sample comprised 26,465 lncRNAs from the PLncDB v.2.0 database was used to estimate the *K*_*n*_ substitution rate in lncRNA. Reference lncRNA sequences were aligned to 150,592 lncRNAs from the pan-transcriptome, accounting for 7% of all identified lncRNAs (see Supplementary Table S4 for details). Of these, 583 were core, 5,436 shell, and 1,492 cloud. Consequently, 144,580 substitutions were obtained for non-coding transcripts.

**Fig. 5:**
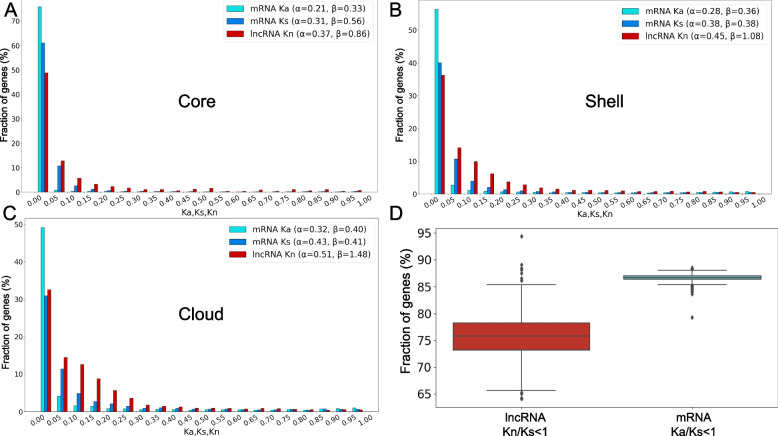
**A**, **B**, **C** Distribution of core/shell/cloud genes by *K*_*a*_*, K*_*s*_ and *K*_*n*_ values. X-axis corresponded to intervals of *K*_*n*_*/K*_*s*_*/K*_*a*_ and Y-axis to percentage of genes within each interval. **D** Proportion of conserved genes with *K*_*a*_*/K*_*s*_ < 1 (mRNA) and *K*_*n*_*/K*_*s*_ < 1 (lncRNA) per library. Y-axis corresponded to the percentage of conserved genes within each library

Histograms of *K*_*s*_, *K*_*a*_ and *K*_*n*_ distributions across the three pan-transcriptome components, along with estimated gamma distribution parameters (*α*, *β*) for their approximation, are presented in Fig. 5A-C. These histograms demonstrate that lower *K*_*a*_ and *K*_*s*_ values correspond to bins with higher values compared to *K*_*n*_, whereas *K*_*n*_ exhibits elevated frequencies at higher substitution rates. This indicates greater nucleotide substitution rates in lncRNA relative to both non-synonymous and synonymous substitutions in mRNA. These patterns are further quantified through the shape parameter *α*, which systematically increases in the order *K*_*a*_ < *K*_*s*_ < *K*_*n*_ across all pan-transcriptome components (core, shell, cloud). This progression reveals that the rate of the nucleotide substitution accumulation is higher in lncRNA than in synonymous sites of mRNA. Moreover, the inequality *α*(*K*_*a*_) < *α*(*K*_*s*_) universally holds for mRNA, which is generally typical for mRNA substitutions under stabilizing selection regimes.

A key observation in Fig. 5A-C is systematic variation in *α* across pan-transcriptome components. For all substitution rates (*K*_*s*_*, K*_*a*_*, K*_*n*_), *α* increases consistently in the order core < shell < cloud. This indicates weaker selective constraints in cloud-associated sequences for both mRNA and lncRNA.

The average *K*_*a*_*/K*_*s*_ ratio and the proportion of conserved transcripts (*K*_*a*_*/K*_*s*_ < 1) were evaluated for protein-coding genes in each maize line. Similarly, the *K*_*n*_*/K*_*s*_ ratio for each lncRNA transcript and the proportion of conserved transcripts (*K*_*n*_*/K*_*s*_ < 1) were evaluated using lineage mean *K*_*s*_ as the denominator for all lncRNAs. Boxplots in Fig. 5D display the distribution of conserved gene fraction for the mRNA and lncRNA pan-transcriptomes. The mean proportion of conserved lncRNA genes (76%) is lower compared to coding sequences (87%). Furthermore, the distribution of conserved lncRNA genes exhibits substantially greater dispersion than that of conserved mRNA genes. These results highlight higher variability in maize lncRNA-encoding genes relative to protein-coding genes.

### Diversity of lncRNAs classified by genomic location

**Fig. 6:**
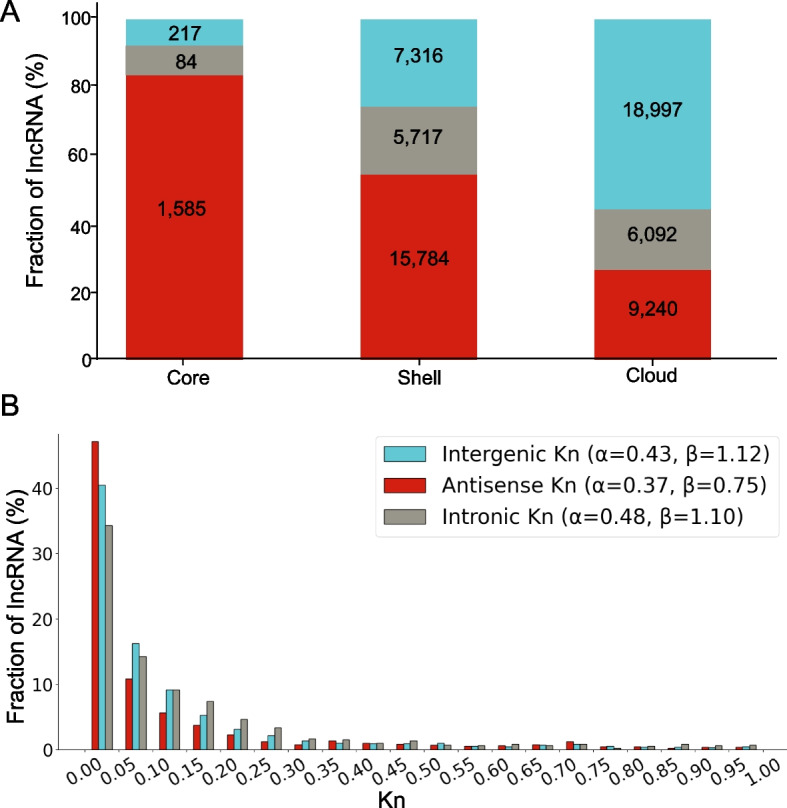
Analysis of lncRNA classes in the pan-transcriptome. **A** Distribution of lncRNA classes across pan-transcriptome components. X-axis corresponds to pan-transcriptome components (core/shell/cloud) and Y-axis to percentage of lncRNAs belonging to each component. **B** Distribution of lncRNA classes by nucleotide substitution rate (*K*_*n*_). X-axis corresponds to *K*_*n*_ value intervals and Y-axis to percentage of genes per class within each interval

ICannolncRNA program [31] allowed classifying lncRNA aligned to the reference genome into three types relative to genomic context: antisense, intergenic, and intronic. Analysis of each type of sequences across pan-transcriptome compartments and substitution rates (*K*_*n*_) is shown in Fig. 6.

Antisense lncRNAs predominantly occur in the pan-transcriptome core (~ 80%). Their proportion decreases to 50% in the shell and becomes smaller in the cloud (~ 25%). Intergenic and intronic lncRNAs exhibit inverse patterns: their fraction increases along in the core, shell and cloud parts. The most pronounced rise observed in intergenic lncRNAs (~ 10% in core to > 50% in cloud). Intronic lncRNAs show similar but less marked trend. Thus, intergenic lncRNAs appear to constitute the most volatile component of the non-coding pan-transcriptome.

Substitution rate comparisons reveal antisense lncRNAs as the most conserved class (Fig. 6B). Their *K*_*n*_ histogram is skewed toward lower values, with the lowest gamma distribution shape parameter *α* among the three types (*α* = 0.37). Notably, intronic lncRNAs exhibit the highest mean *α* (0.48), exceeding that of intergenic lncRNAs (*α* = 0.43). This suggests stronger selective constraints on intergenic lncRNAs compared to intronic lncRNAs, potentially indicating greater functional significance in intergenic lncRNAs.

## Discussion

Analysis of lncRNA evolution has shown that these molecules undergo rapid evolutionary turnover, resulting in only a small proportion of sequences being comparable across species. In this study, comparative analysis of maize lncRNAs is performed within the pan-transcriptome framework, enabling assessment of their sequence diversity, identification of variability patterns across numerous sequences, and comparison with mRNA variability characteristics. Crucially, transcript identification, for both mRNAs and lncRNAs, is performed using identical data and software pipelines, ensuring maximally rigorous comparison.

RNA-seq libraries from the work by Hirsch et al. [37] were used. They were obtained from the seedling samples of 503 maize inbred lines. This number of samples allows for the capture of substantial transcriptome diversity. However, the sequencing depth for these libraries is relatively low, not exceeding 20× for the majority. Hirsch et al. [37] employed two approaches for assembling the pan-transcriptome. The first approach involved pooling reads from all libraries. Some of these reads were mapped to the reference genome, while the remaining reads were used for de novo assembly (using the Velvet and Oases programs). The de novo assembly resulted in the reconstruction of ~ 100,000 transcripts, forming ~ 30,000 representative transcript assemblies (RTAs). Of these, ~ 22,000 exhibited high similarity to protein-coding genes in the reference genome, while ~ 8,000 were novel sequences. The second pan-transcriptome assembly strategy was based on individually assembling the 366 inbred lines with the highest coverage and subsequently merging the transcripts into RTAs. This assembly approach yielded ~ 2,200,000 transcripts, consolidated into ~ 750,000 RTAs. These data were used to assess the type of pan-transcriptome (open or closed). In our pipeline, we utilized a combined de novo transcript assembly for all libraries using three methods (Trinity, TrinityGG and rnaSPAdes). This approach is similar to the second one described in Hirsch et al. [37]. For mRNA, approximately 14,000,000 transcripts and ~ 250,000 RTAs were obtained. This quantity falls between the conservative and expanded estimates of RTA numbers reported in [37] and is closer in order of magnitude to the latter. However, an exact comparison of the transcriptome assembly results between the two studies appears hardly feasible.

In agreement with previous studies [25, 79], we obtained that the composition of lncRNAs exhibits significantly higher variability among maize inbred lines compared to mRNAs. This is evidenced by a pan-transcriptome size nearly three times larger (see Fig. 1, Fig. 2). Transcript-set size analyses indicated the ability to cover ~ 95% of the mRNA pan-transcriptome with a random sampling of 25 individuals and 99% with 93 individuals (see Fig. 2A). Interestingly, these values are close to estimates obtained by Gui et al. [35], where saturation to 95% of genes in the maize pan-genome is achieved with 27 samples, and to 99% of genes with 99 samples. Our results show the closed type of the protein coding pan-transcriptome in maize inbred lines. This is consistent with the results of Hirsch et al. [37] and with findings from the study of the maize pan-genome across 721 individuals, which also demonstrates its closed nature [35].

In contrast, 95% of transcripts in the lncRNA pan-transcriptome can be represented by 308 inbred lines, which is 12 times higher than the corresponding value for mRNA (Fig. 2A, Sect. "Pan-transcriptome structure analysis"). This further demonstrates the high variability of lncRNAs compared to mRNAs. It is also noteworthy that the saturation of the lncRNA pan-transcriptome occurs more slowly with increasing numbers of inbred lines than for mRNA. These results are in good agreement with the data obtained by Kornienko et al. [80]. They analyzed lncRNAs in transcriptomes from 499 *Arabidopsis thaliana* accessions. Results demonstrated that unlike protein coding genes, the number of lncRNAs strongly depended on the sample size and showed no sign of saturating even with 460 accessions. Here, according to power law estimates [40], the maize lncRNA pan-transcriptome is determined as closed, similar to the mRNA pan-transcriptome, despite its lower saturation rate (see Fig. 2). The question of the type of the lncRNA pan-transcriptome is of importance to determine possible mechanisms of ePAVs. Kornienko et al. [80] showed, that the expression variability of lncRNAs in Arabidopsis determined mainly by the epigenetic patterns across accessions. This implies that the total number of lncRNA loci in genomes of various accessions is almost the same. In this case, the lncRNA pan-transcriptome is expected to be closed.

The core size of the mRNA pan-transcriptome constitutes 8% of the total number of cluster centroids (~ 20 thousand transcripts). This is comparable to the number of known protein-coding genes in the reference genome (~ 30 thousand; [81]). Studies of protein coding gene conservation across different pan-genomes have revealed that core gene clusters range from 23 to 89%, shell gene clusters from 10 to 53%, and cloud gene clusters from 0.1% to 43% [82]. For example, in *Brachypodium distachyon* pan-genome, on average, individual lines were composed primarily of core genes (73%), while only 27% were assigned to the shell or cloud categories [83]. In contrast, in barley pan-genome the cloud component was slightly larger than the core component [84]. Nevertheless, similar to our data, in most cases the proportion of cloud gene clusters has been found to be lower than that of core or shell categories, with their distribution varying considerably between individuals [82].

In contrast, the lncRNA pan-transcriptome demonstrated a predominance of sequences from the cloud component (see Fig. 3). This is in agreement with the rapid evolutionary turnover of lncRNAs and predominance of species-specific sequences among lncRNAs [23–25]. Our results are also in agreement with studies of lncRNA in the population of *A. thaliana* [80], demonstrated that lncRNA expression patterns are largely variable between accessions with half of all lncRNAs being expressed in 1 accession while being off in another.

Substantial differences in nucleotide sequences for lncRNAs compared to mRNAs within the maize pan-transcriptome are observed, in accordance with previous studies in plants and animals [12, 24, 85, 86]. Estimates of Tajima's *D* parameter values in our study are skewed toward positive values for both mRNAs and lncRNAs. For protein-coding genes, our results differ significantly from the Tajima's *D* distribution obtained in the analysis of the maize pan-genome across 721 samples (which shifted towards negative values) [35]. However, it should be noted that unlike in the study by Gui et al. [35], data from Hirsch et al. [37] represent maize inbred lines, which characteristic is a deficit of low frequency alleles relative to expectation due to a possible population bottleneck, population subdivision, or balancing selection [87]. We also note that this test demonstrates differences in lncRNAs and mRNAs nucleotide substitutions: the distribution of Tajima's *D* for lncRNAs is shifted toward higher values.

Estimates of rates of nonsynonymous and synonymous substitutions in mRNAs, as well as substitutions in lncRNAs, also revealed greater sequence variability in lncRNAs: the *α* parameter of these distributions increases in the series *K*_a_ < *K*_s_ < *K*_n_, indicating an increased rate of nucleotide substitutions. Furthermore, the substitution rate *K*_n_ in lncRNA sequences exceeds even the neutral substitution rate *K*_s_ in mRNAs. Interestingly, we observed the same trend of increasing substitution rates for the *α* parameter across three substitution types depending on pan-transcriptome compartment: *K*_core_ < *K*_shell_ < *K*_cloud_. This reflects the evolutionary conservation of sequences belonging to different pan-transcriptome compartments, in agreement with the current knowledge [33, 35, 36].

In agreement with previously established data [25, 31], analysis of the lncRNA belonging to three different types (antisense, intronic and intergenic) demonstrated highest sequence conservation for antisense lncRNAs, intermediate conservation for intergenic lncRNAs and greatest variability in intronic lncRNAs. Intergenic lncRNAs, however, demonstrate higher ePAV diversity: they are most frequently represented in the cloud component of the pan-transcriptome. In protein coding pan-transcriptomes most of the cloud genes belong to low-occupancy and low-expression sequences [82, 83]. Their functions are commonly linked to environmental responses and defense mechanisms, including receptor and antioxidant activity, gene regulation, and signal transduction [32]. They also tend to be evolutionarily younger than core genes [32]. This suggests that intergenic lncRNAs may contribute to the regulation of plant stress responses and environmental adaptation as well. Interestingly, several studies have shown that intergenic lncRNAs are related to plant stress response [88–91].

Pan-transcriptome analysis provides consistent basis for examination a range of RNA sequence variability characteristics in maize transcriptomes using unified bioinformatics approaches for both mRNAs and lncRNAs. Here, consistent evolutionary patterns were identified: substantially greater compositional diversity of lncRNAs compared to mRNAs, elevated substitution rates in lncRNAs, and the more conserved nature of antisense and intergenic lncRNAs relative to intronic lncRNAs.

Several studies have previously noted that lncRNA analysis can inevitably be influenced by multiple factors: library quality, genome assembly and annotation quality [24], and the choice of software for lncRNA identification and annotation [92]. Research on pan-genome reconstruction methods using different sequence clustering approaches has further demonstrated that the choice of assembly strategy and clustering methodology can affect structural property estimates [93]. In the current work, the sequencing depth for RNA-seq data is quite low. This important limitation may result in the underrepresentation of low abundance transcripts in the pan-transcriptome. Low sequencing depth may affect the completeness of both mRNAs and lncRNAs sets. However, this factor has a greater impact on non-coding transcripts due to their lower expression [24]. The second reason for possible incompleteness of both pan-transcriptomes is the usage of whole-seedling tissue at only the first leaf stage. It is well known that lncRNA expression is specific to particular tissues and developmental stages [94, 95]. Therefore, some lncRNA transcripts can be missed when using one or few development stages/tissues/conditions. For example, Kornienko et al. [80] demonstrated that while the number of lncRNA loci always increased with more accessions, the number of tissues used mattered even more.

In addition to low sequencing depth and one development stage for the RNA-seq experiments, expression threshold may affect the estimates of the pan-transcriptome size and structure. Here, we applied the same threshold for both mRNA and lncRNA transcripts (TPM > 1). This threshold is generally accepted to remove noise for mRNAs [96–98]. However, for most lncRNAs expression level is lower than for mRNAs [24]. In this regard, some authors applied lower values of the expression threshold to filter lncRNA noise, 0.3–0.5 TPM [80, 99]. Other authors applied TPM > 1 rule to select lncRNAs [100–102], justifying this choice as a way to reduce higher levels of noise in lncRNA data. In our study, we applied the latter criteria taking into account low sequencing depth of the RNA-seq libraries, which may lead to increased noise. The threshold for sequence similarity applied for clustering mRNA and lncRNA sequences is another source of ambiguity in our analysis. We used different values for mRNA (80% identity) and lncRNA (70% identity) obtained empirically taking into consideration unequal rates of amino acid and nucleotide substitutions. Nevertheless, RNA-seq protocols, data processing methods, and sequence analysis pipelines are identical for both mRNAs and lncRNAs. This ensures an unbiased comparative assessment of lncRNA and mRNA diversity based on pan-transcriptomic data.

## Conclusion

Analysis of RNA-seq libraries from 503 maize inbred lines enabled the assessment of sequence diversity and evolutionary patterns of maize lncRNAs within the pan-transcriptome framework and their comparison with analogous mRNA characteristics. Both pan-transcriptomes are closed; however, the lncRNA pan-transcriptome comprises a greater number of sequences, the majority of which fall into the cloud component. This demonstrates the high lineage-specificity of the lncRNAs across maize inbred lines. Analysis of nucleotide substitutions revealed that lncRNAs in the maize pan-transcriptome exhibit higher diversity compared to mRNAs. This characteristic of lncRNAs is further supported by their elevated substitution accumulation rate, exceeding even the rate of neutral substitutions in mRNAs. Comparison of evolutionary characteristics among the three lncRNA types showed that antisense lncRNAs are the most conserved in terms of both nucleotide substitution rates and compositional stability, intronic lncRNAs display the highest mutation rate, while intergenic lncRNAs exhibit the greatest sequence diversity and highest line specificity.

Thus, employing the pan-transcriptome concept enabled a deeper analysis of the features underlying intraspecific lncRNA variability in maize.

## Supplementary Information

Supplementary Material 1.

Supplementary Material 2.

## Data Availability

All data generated or analyzed during this study are included in this published article and its supplementary information files.

## Abbreviations

lncRNA: Long non-coding RNA
PAV: Presence/absence variation
ePAV: Expression presence/absence variation
