# Supplementary material for: Diversity of lncRNAs in the pan-transcriptome of maize inbred lines
Source: BMC Genomics. 2026 Jan 3;27:1. doi: 10.1186/s12864-025-12242-0 (PMC12764133; doi:10.1186/s12864-025-12242-0)
Supplement: Supplementary file 2 — Supplementary Material 2. [file 12864_2025_12242_MOESM2_ESM.pdf]

Supplementary tables and figures

Table S1. Statistics of maize transcriptomes assembly using three different methods.

Assembly statistics	TrinityGG	Trinity	rnaSPAdes
Average contig length	942	864	592
Total number of transcripts	882,370	21,646,755	44,149,357
GC%	47	51	51
N50	1,834	1,514	1,475

Table S2. Statistics of the separation of maize transcriptomes into protein-coding and non-coding transcripts. Percentages in parentheses show the fraction of sequences remaining compared to the previous step.

Processing stages	TrinityGG	Trinity	rnaSPAdes
Number of input sequences	896,138	21,646,755	44,149,357
Filter by sequence length	895,964 (99%)	21,646,755 (100%)	31,740,702 (71%)
Non-coding	647,415(73%)	12,951,969 (59%)	19,194,063 (60%)
Protein-coding	248,549 (27%)	8,694,786 (40%)	12,546,639 (40%)

Table S3. Results of the filtering steps for protein-coding sequences. Percentages in parentheses show the fraction of sequences remaining compared to the previous step.

Processing step	TrinityGG	Trinity	rnaSPAdes
Protein-coding transcripts predicted by ICAnnolncRNA	248,549	8,694,786	12,546,639
Removal of duplications (100% sequence match)	248,549 (100%)	8,694,786 (100%)	12,546,639 (100%)
Application of Evidential Gene pipeline	327,250 (131%)	9,763,618 (112%)	13,884,606 (111%)
Filter by expression (TPM<1)	296,259 (91%)	9,256,410 (95%)	13,091,293 (94%)
Contamination filter (Univec, viral sequences)	286,216 (97%)	8,794,070 (95%)	12,428,199 (95%)
Filter sequences similar to non-cereal proteins	174,069 (61%)	7,325,503 (83%)	10,296,162 (83%)

Table S4. Results of the filtering steps for non-coding sequences after alignment to the reference genome. Percentages in parentheses show the fraction of sequences remaining compared to the previous step.

Filtering steps	TrinityGG	Trinity	rnaSPAdes
Non-coding transcripts identified by ICAnnolncRNA	647,415	12,951,969	19,194,063
Aligned to reference genome by ICAnnolncRNA	641,501 (99%)	4,593,888 (35%)	18,269,583 (95%)
Filtering transcripts aligned to the same locus by ICAnnolncRNA	330,284 (51%)	324,813 (7%)	784,254 (5%)
Filtering transcripts overlapped with TEs	107,239 (32%)	191,130 (58%)	422,316 (53%)
Filtering transcripts overlapped with exons in the same strand	106,468 (99%)	155,009 (81%)	239,005 (56%)

Table S5. Results of the filtering steps for non-coding sequences that did not align to the reference genome. Percentages in parentheses show the fraction of sequences remaining after filtering, relative to the previous step.

Filtering steps	TrinityGG	Trinity	rnaSPAdes
Non-coding transcripts predicted by ICAnnolncRNA	647,415	12,951,969	19,194,063
Unmapped	5914 (1%)	8,358,081 (65%)	924,480 (5%)
Filtering sequences similar to TEs (>80% Score)	5860 (99%)	7,544,003 (90%)	919,019 (99%)
Filtering matches to protein-coding sequences (100% coverage)	5856 (99%)	6,897,352 (91%)	887,528 (96%)
Filtering matches to known maize ncRNA sequences (100% coverage)	5856 (100%)	6,841,977 (99%)	884,711 (99%)
Redundancy removal (vsearch)	4558 (77%)	1,039,773 (15%)	474,113 (53%)

Table S6. Results of the filtering steps for all lncRNAs. Percentages in parentheses show fraction of sequences remained in comparison with previous step.

Filtration stages	TrinityGG	Trinity	rnaSPAdes
lncRNA predicted by ICAnnolncRNA (aligned to the reference genome)	106,468	155,009	239,005
lncRNA predicted by ICAnnolncRNA (not aligned to the reference genome)	4,558	1,039,773	474,113
Merging	111,026	1,194,782	713,118
Contamination filter (Univec, viral sequences)	110,320 (99%)	1,193,801 (99%)	711,487 (99%)
Removal of duplications (100% sequence match)	110,123 (99%)	1,193,801 (100%)	703,672 (99%)

Table S7. Statistics of pan-transriptome assembly of coding (mRNA) and non-coding (lncRNA) parts.

Processing step	mRNA	lncRNA
Meta-assembly	17,795,733	2,007,596
Deduplication	14,392,809	2,007,596
Clustering	245,436	595,198

Table S8. Statistics for the distribution of nucleotide diversity (π) for mRNAs and lncRNAs in the maize pan-transcriptome.

Statistics	mRNA	lncRNA
Median	1.58×10^{-5}	1.94×10^{-5}
Lower quartile Q1	8.31×10^{-6}	9.33×10^{-6}
Upper quartile Q3	3.26×10^{-5}	4.70×10^{-5}
Minimum	6.67×10^{-7}	9.00×10^{-7}
Maximum	0.09×10^{-3}	0.02×10^{-2}
Interquartile Range	2.42×10^{-5}	3.77×10^{-5}
Standard deviation	4.69×10^{-5}	4.46×10^{-5}

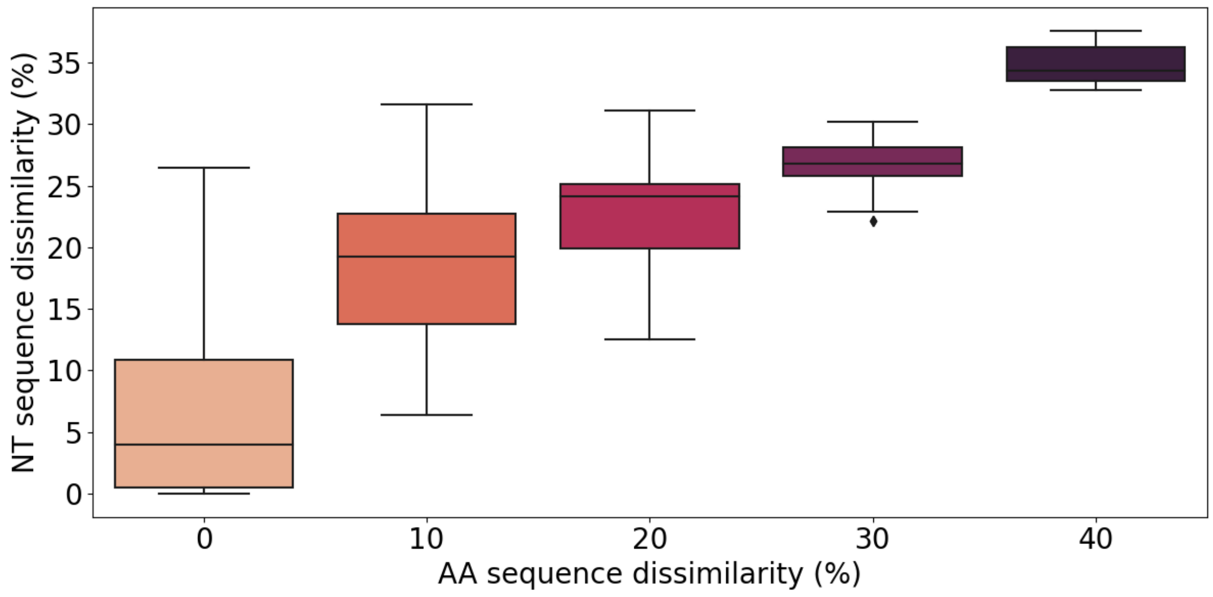

Figure S1. Boxplots for distributions of dissimilarity in pairs of aligned mRNAs (Y axis) at the different values of dissimilarity for amino acid sequences translated from them (X axis).

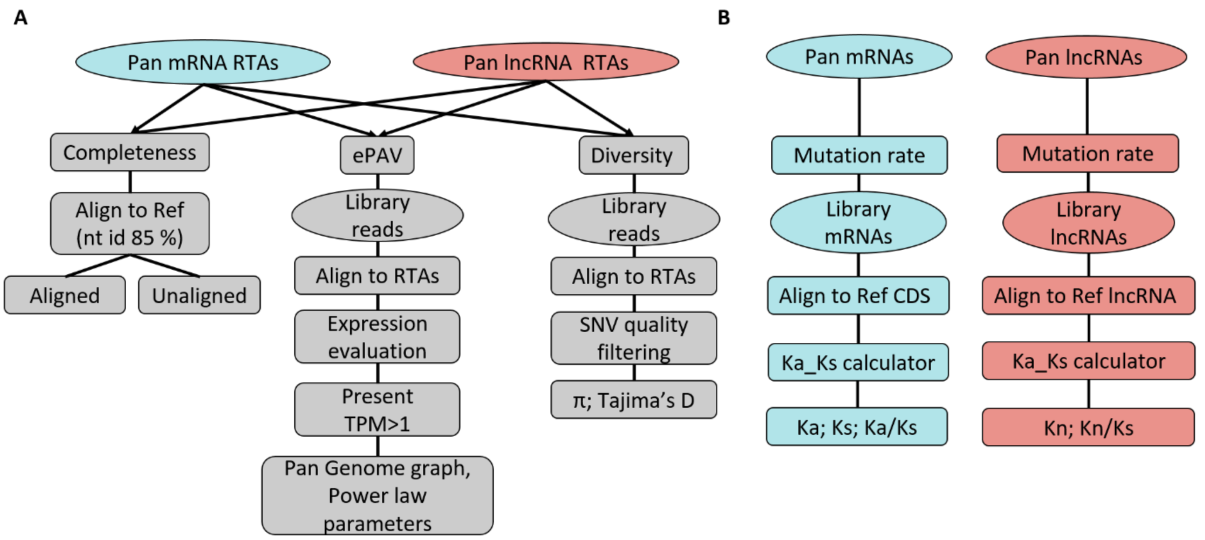

Figure S2. Pan-transcriptome analysis pipeline. Gray color denotes analysis stages common to both mRNA and lncRNA. A). Pan-transcriptome SNP analysis. B). Analysis of synonymous and non-synonymous substitutions.

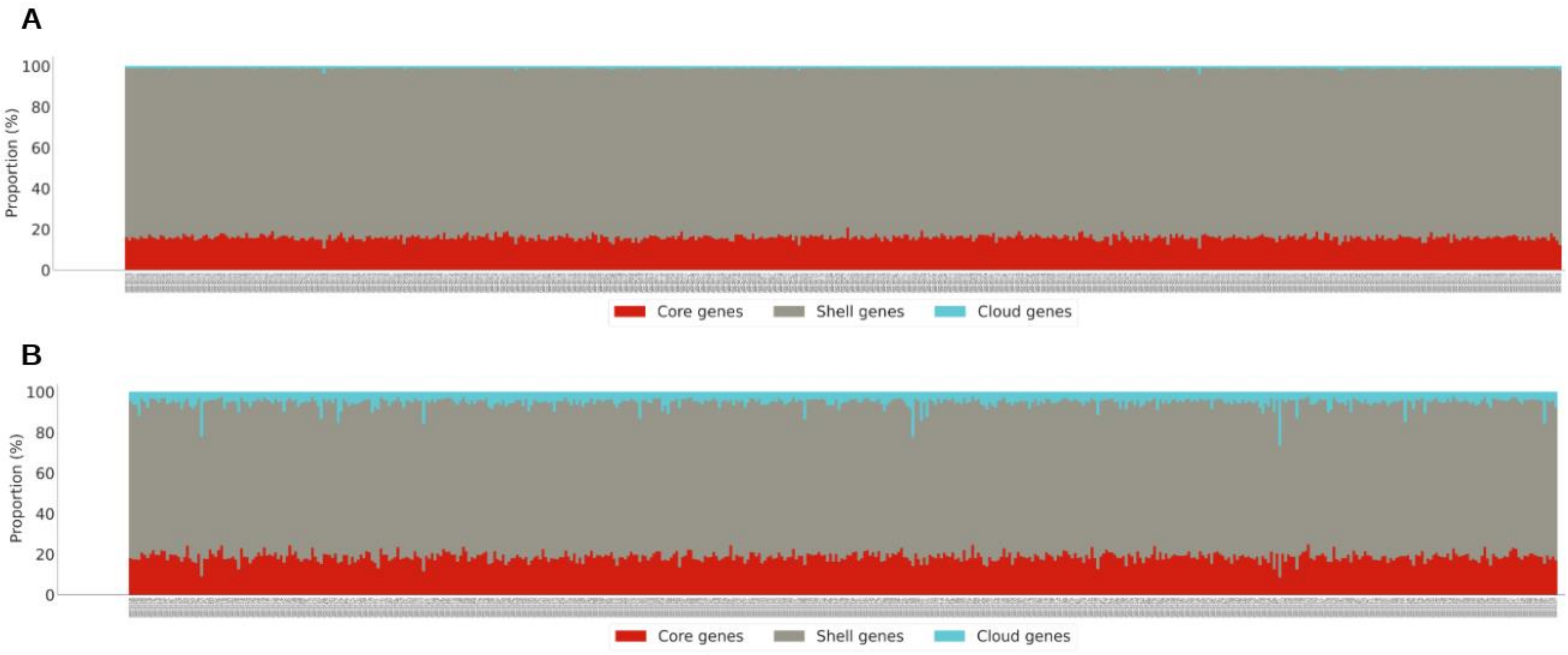

Figure S3. Fraction of genes belonging to the core, shell, cloud parts in maize inbred lines. A). mRNA pan-transcriptome; B). lncRNA pan-transcriptome. The X-axis shows 503 maize libraries, the Y-axis shows the percentage of genes belonging to a pan-transcriptome part.
